# Cognitive Behavioral Therapy of Patients with Somatic Symptoms—Diagnostic and Therapeutic Difficulties

**DOI:** 10.3390/jcm10143159

**Published:** 2021-07-17

**Authors:** Agata Orzechowska, Paulina Maruszewska, Piotr Gałecki

**Affiliations:** Department of Adult Psychiatry, Medical University of Lodz, Aleksandrowska 159, 91-229 Lodz, Poland; paulina.zuchowicz@stud.umed.lodz.pl (P.M.); piotr.galecki@umed.lodz.pl (P.G.)

**Keywords:** psychosomatic, somatoform disorders, functional disorders, somatization, somatic diseases, depression, anxiety disorders, cognitive behavioral therapy

## Abstract

In every somatic disease we can find a psychological element, just as it is not uncommon for numerous physical symptoms to occur in a mental disease. Nowadays, the patient is no longer just the “owner” of the sick organ but is considered and treated as a “whole”. The interpenetration of somatic manifestations with mental health problems forces patients who experience subjective suffering, including mental suffering, from current symptoms to visit specialists from different fields of medicine, and their treatment does not bring about any improvement. Cognitive behavioral psychotherapy (CBT) is one form of therapy that attempts to respond to the needs of an increasing—in recent years—number of patients who demonstrate somatic disorders of a multifaceted nature. The co-occurrence of physical and mental disorders repeatedly makes it impossible to determine which symptoms were the cause and which were the effect; hence, it is difficult to establish clear boundaries between the categories of these disorders and diseases. The therapist, to whom the patient with somatic diseases is eventually referred, may be faced with a diagnostic dilemma, the solution of which will give direction to further psychotherapeutic work. The common feature of this group of patients is a strong focus on physical ailments, while omitting or almost completely ignoring the psychological factors involved. The purpose of this paper is to present the causally diverse circumstances in which a patient with physical symptoms needs diagnosis and therapeutic support from the perspective of a cognitive behavioral approach.

## 1. Introduction

The number of patients who report to a family doctor with somatic disorders caused by psychological reasons accounts for about 30% of all those who receive medical assistance. Somatic disorders are some of many forms of “expressing” abnormalities in the mental sphere, and mental disorders may be accompanied by somatic symptoms. Psychosomatic disorders and functional disorders are common manifestations in general medical practice in both primary and secondary health care. They cause clinical problems for practitioners due to their uncertain nature and lack of effective treatment. They also provoke difficulties when classifying them using current taxonomic systems. On the other hand, a chronic somatic disease can also lead to or accompany mental disorders [1,2,3].

One of the consequences of the correlation between the somatic and mental spheres is that the patient who shows predominantly somatic signs, having a multifaceted background in the clinical picture, is eventually referred to a psychotherapist. Depending on the diagnosis made or the ongoing process of searching for it, these patients can be classified into several groups. The framework of these groups is conventional and fluid because it is often difficult to confirm which symptoms occurred earlier [4,5]. The first group are patients with a diagnosis of a chronic or incurable somatic disease that can lead to adaptive disorders, such as a depressive or anxiety reaction. They can also be people with so-called functional or physical symptoms of a psychological background, without a confirmed organic disease (e.g., irritable bowel syndrome). Another group includes patients who have a mental disease, which is accompanied by somatic manifestations (headaches, muscle aches, feeling of fatigue in depression), being a part of the psychiatric diagnosis. Finally, there is a population of people reporting to a psychotherapist with somatic symptom disorders (traits of somatization, hypochondria, i.e., disorder with fear of health, persistent psychogenic pain and other neurotic disorders with somatic symptoms) [5,6].

The extensiveness of the subject matter presented in this article does not allow the description of all possible causes for why somatic signs motivate a patient to contact a psychotherapist. The authors focus on selected groups of disorders and diagnostic and therapeutic problems in the context of cognitive behavioral therapy. Publications reviewing the research on therapeutic methods used in cases of co-occurrence of bodily and psychological symptoms point to various therapeutic interventions. Cognitive behavioral therapy is indicated as the most commonly used therapy of choice and enjoys a large number of reports of efficacy, primarily in patients with somatization disorders [7]. There are no reports in the literature presenting this problem in a comprehensive manner, and the models of cognitive behavioral therapy and proposed protocols usually do not exhaust the possibility of working with patients with somatic manifestations that have a diverse background [6,8].

The authors of this paper analyzed the literature on psychosomatic and somatopsychic relationships in the medical care of patients. The starting point draws attention to the difficulty of making an unambiguous diagnosis and identifying effective treatment of patients reporting the co-occurrence of physical and psychological symptoms. This clinical problem, mainly related to planning the therapeutic process, is discussed in the context of cognitive behavioral therapy.

## 2. The Relationship between Psyche and Body—Why a Patient with Somatic Symptoms Gets Psychotherapy

The relationship between psyche and body is multi-directional. In literature, it has long been defined as a psychosomatic relationship. In conjunction with biological factors, psychosocial factors influence the formation of disorders and diseases both directly and indirectly. Doctors often face the problem of finding the primary cause of the symptoms presented by the patient, and the patient is unable to define the timing of the relationship of the somatic problems with the mental ones. It is difficult in many situations to determine whether emotional distress causes somatic symptoms or somatic distress causes emotional symptoms, or whether they interact. On the one hand, in cases that are difficult to diagnose and difficult for the medical team to recognize, there is a tendency to mistakenly explain the physical signs as being of a psychiatric origin. As a result, many patients with complex and poorly understood illnesses who receive inadequate evaluation of their condition are unduly referred to psychiatrists. On the other hand, often a patient goes a long way to perform a series of physical examinations to find the cause of problems before finally going to a psychiatrist or psychologist [9,10].

Diagnostic difficulties in differentiating the somatic and psychological causes of the complaints reported by patients generate a number of negative effects, such as excessive costs of tests and delays in starting the treatment process, which can be a source of stress—both for the doctors who perceive this type of patients as frustrating and demanding but above all for the patients themselves who report dissatisfaction, disbelief and disregard by the doctors [11].

Chronic somatic diseases and disorders entail a fundamental and usually unsuccessful change in human life in the form of persistent ailments causing suffering and limitations in fulfilling important social roles—both family-related and professional. Psychological factors form the basis of any work with the patient with a chronic somatic disease, and the effectiveness of therapy can be very high and has recently been more widely appreciated [12].

Parallel with a somatic disease, depending on its course, prognosis or mental stress, psychopathological syndromes may develop (e.g., depression or anxiety disorders in a patient after myocardial infarction or suffering from cancer). Emotional processes often form part of the picture of a somatic disease or are a reaction to negative consequences of the disease. Because of the negative impact of emotional processes on the health condition of the patient with a chronic somatic disorder, in many situations, doctors of various specialties cannot offer sufficiently effective treatment. Mental disorders in somatic diseases, which are their consequence, can occur at any stage of the somatic disease. Bodily manifestations can then be amplified by experiencing unpleasant emotional states (e.g., anxiety symptoms) and by secondarily concentrating strongly on them, which may complicate the course of the disease. The process of the vicious circle is initiated, which maintains the physical problems and fear of the disease [13,14,15].

Psychotherapeutic assistance will be sought by patients presenting mental disorders who have syndromes of somatic disorders in their clinical picture. Such disease entities include depression and anxiety disorders, the signs of which (constituting diagnostic criteria) include physical problems. Due to physical problems that come to the fore, patients with such a diagnosis are often referred to a family doctor instead of a psychiatrist. Somatic symptoms are so intense that they mask mental disorders, suggesting the presence of a somatic disease. Furthermore, in mental illnesses there is a greater tendency to somatization, i.e., experiencing emotions as somatic disorders. States of reduced mood or anxiety can also be a source of physical ill health [2,16].

Within the group of mental disorders, special attention should be paid to somatic symptom disorders that may present themselves as a diagnostic problem in the offices of family doctors and internists. This diagnostic category includes somatization disorders, undifferentiated somatic symptom disorders, hypochondriacal disorders, somatoform autonomic disorders (dysfunctions), persistent psychogenic pain and other types of somatoform pain [2,17,18].

A large group of patients with psychosomatic disorders are people who have had traumatic experiences in the past. It has been shown that trauma survivors are more than twice as likely to develop psychosomatic disorders compared with the general population, and if the trauma led to the development of PTSD (post-traumatic stress disorder), then this relationship is even more evident. Chronic fatigue syndrome develops most often, although the development of disorders such as irritable bowel syndrome or fibromyalgia is also frequent. The etiology of these disorders in cognitive psychology indicates the role of distorted interpretations arising from traumatic experiences. The experience of trauma can determine the way future potentially threatening situations or stimuli are interpreted, including physical symptoms. This tendency to distorted attributions can then lead to avoidance of some situations or catastrophic interpretations of symptoms. For example, a person with fibromyalgia may experience significant anticipatory anxiety related to the likelihood of future pain, which may in effect isolate or reduce activity levels, potentially leading to greater pain susceptibility and poorer psychosocial functioning. Trauma separates the cognitive sphere from the affective sphere. Many patients suffer from physical symptoms that are caused by dissociation. This means that painful experiences that could not be mentally integrated have become disconnected from the psyche, having an impact on somatics [19].

Eating disorders are another group of psychosomatic disorders related to trauma. Early childhood trauma, and especially the experience of violence, is important in the development and maintenance of eating disorders in childhood, adolescence and adulthood. A recent meta-analysis of the available literature showed that the frequency of experiencing violence in the group of people with eating disorders is significantly higher than in the general population and in the group of patients with other mental disorders [20]. Theoretical approaches explaining the causes of these disorders indicate that eating disorders are a form of autoaggression that helps the victim to work through the experienced trauma and return to balance [21].

Characteristic features of this group of patients are strong concentration of the patient on the somatic disorders without a serious physical disease confirmed, lack of control over the experienced somatic symptoms and the presence of psychological factors that may exacerbate these manifestations, which can suggest mental disorders. Patients are subject to a number of diagnostic tests and various methods of treatment because the symptoms due to which they experience suffering are concentrated around bodily signs from different groups. Such problems can be variable and cause subjective perception of pain and, consequently, lead to mental discomfort. However, their persistence cannot be explained by any detectable somatic disease. The patient rejects the assurances of medical personnel that there is no adequate reason for such complaints [1,6,14,15,18].

The psychotherapist to whom the patient with somatic symptoms is referred is put in a situation of a diagnostic dilemma, the solution of which will give direction to further therapeutic work with the patient. It is difficult or sometimes impossible to differentiate between somatic symptoms resulting from a somatic disease and somatic symptoms being manifestations of functional syndromes associated with mental disorders—all the more so when the patient referred to psychotherapy has an ambiguous history of the diagnostic process and does not accept the decision to report to a psychotherapist and when his or her motivation is low. The psychotherapist is also fully responsible for confirming or ruling out the presence of an abnormal somatic condition by conducting an interview regarding somatic diagnosis before undertaking psychotherapy [14,22].

## 3. Cognitive Behavioral Therapy in the Treatment of Patients with Somatic Symptoms

Observing the importance and place of psychotherapy in medicine over the course of several decades, there has been a clear increase in the interest in and significance of this field of knowledge, as well as an increase in the number of psychotherapists working with somatic patients. Regardless of the primary cause of somatic symptoms, people suffering from these ailments are the target group (increasingly more numerous) of people taking advantage of, among others, cognitive behavioral psychotherapy [4,8].

Meta-analyses indicate that psychotherapy in the cognitive behavioral approach in patients suffering from somatoform disorders significantly reduces the intensity of somatic complaints and the signs of anxiety and depression and improves their physical functioning. The best results were obtained during psychotherapy lasting longer than 12 sessions. These positive effects lasted from three months to one year. It was shown that group therapy contributed most to minimizing somatic symptoms, while individual psychotherapy was most effective in reducing signs of depression and anxiety [23].

Furthermore, for patients with chronic somatic illness, CBT has proven to be an extremely effective form of non-medical treatment. The use of CBT-based techniques with cancer patients has been shown to be highly effective in reducing negative emotions and thoughts caused by cancer diagnosis and treatment. This psychotherapy is helpful in minimizing pain and coping with side effects of chemotherapy and/or radiation therapy. It has also been shown to foster a stronger sense of control over one’s feelings and behaviors related to coping with the disease [24].

Cognitive behavioral therapy (CBT) is a current of psychotherapy that has evolved from behaviorism to cognitive therapy to a whole group of cognitive behavioral therapies, including, among others, mindfulness or acceptance and commitment therapy. The concept is based on the assumption that a human’s reaction to a given situation is determined by what he or she thinks about the specific event, i.e., what meaning he or she will give to it. The described current assumes that the source of the patient’s problems is a negative and unjustified reaction to difficult situations, which is reflected in thoughts, emotions, physical symptoms and actions [25]. The aim of the therapy is to change the way of thinking and behavior to the extent deemed necessary by the patient. Thoughts are verified on three levels: automatic thoughts, assumptions and core beliefs. The psychotherapist also helps to organize, understand and free the patient from difficult emotions with the use of properly selected tools [26,27].

### 3.1. Chronic Somatic Disease

The scheme of working with a patient with somatic symptoms boils down to the relationship between the somatic sphere, mental sphere and the surroundings of the individual. This perspective corresponds to the biopsychosocial model of health and disease. Regardless of the primary background (somatic, mental, mixed) of the somatic problems, the common feature of this group of patients is reporting physical complaints as dominant complaints during therapy. The formulation of the case, taking into account the patient’s life situation and mechanisms supporting his or her diseases, becomes crucial in cognitive behavioral therapy [15,16].

In the situation of patients with a chronic somatic disease, such as cardiological, neurological or neoplastic disease, it is important to take into consideration a fundamental change involving the need to assume the role of the patient, i.e., change in the hierarchy of objectives and values, as well as the need to modify health habits and behavior in the broadest sense. The patient has to submit to the therapeutic regime that is associated with frequent examinations, hospitalizations or, finally, very burdensome treatment methods. The patient experiences the fear of suffering and death. The professional or family situation of the patient changes. There is a sense of threat and loss of security, loss of control over one’s health and, consequently, a sense of helplessness. Finally, human physical functioning changes. There are restrictions regarding mobility, and there is often disability and disfigurement. All this is reflected in a change in the patient’s self-image. The treatment itself, or rather its side effects, is also important. Patients often experience significant physical discomfort during treatment; psychological reactions caused by the applied treatment may also appear, such as irritability, cognitive dysfunction, consciousness disorders or emotional instability, and even intensified suicidal thoughts [28,29].

The care of patients who are somatically ill in the cognitive behavioral current comes down mainly to the way of thinking about the disease and the possibilities of coping with it, which has a secondary impact on the emotional functioning of the patients and their behavior. Chronic illness affects the patient’s life, changing their mood, behavior and way of thinking about themselves and the world that surrounds them. Thoughts that arise in the situation of illness often have a distorted and maladaptive nature. Since coping strategies are influenced by the image of the disease, great importance is attributed in the model of cognitive behavioral psychotherapy to the processes associated with making meaning of the disease. The normalization of the cognitive content associated with the image of the disease affects stress response regulation, which may determine the actions taken by the patient to cope with the challenges associated with such a difficult life situation [30].

The specifics of working with a somatically ill patient also involve focusing on the patient’s quality of life, reinforcing its good sides, and focusing on the patient’s current problems. It is necessary to adapt working methods to the patient’s abilities and to use short interventions. Cognitive behavioral therapy aimed at changing negative cognitive content and developing new thought habits, conducive to the desired emotions and behaviors, may have a positive effect on changing the mental state of patients with cancer [22,28]. The long-term goal of such psychotherapeutic interactions is to teach the patient how to cope with the disease, to motivate him or her to participate in the process of treatment of the disease and to modify incorrect habits and behavioral patterns, as well as to cope with adaptation difficulties. Psychotherapeutic help for incurable and terminally ill patients most often involves palliative actions, helping them to endure suffering when they are no longer undergoing active forms of therapy [28,29].

Cognitive behavioral psychotherapy of patients who are treated due to a somatic disease and who experience syndromes of mental symptoms (anxiety and depression disorders) refers mainly to models of cognitive affective and anxiety disorders. The therapy allows the patient to create or recreate mental structures that facilitate the internal process of calming down. In the situation of a therapeutic relationship, the patient has the opportunity to experience fearful images of his or her health in a safe way and gain distance and ambivalence to them, as well as to reflect on their meaning and significance. In this way, the interpretation of the experienced symptoms changes, and the patient’s fear of death is diminished, thus reducing the severity of the manifestations [13].

The therapeutic process should not ignore beliefs about the negative impact of anxiety and worry about physical health through repeated and chronic physiological excitation resulting from the experienced negative emotional states [28,30]. During sessions, it is necessary to monitor the mental condition of patients with a chronic somatic disease, assessing all patients for possible mental disorders, specifically those who experienced mental disorders in the past. The therapist can use numerous psychometric tools, e.g., the Beck Depression Inventory, the Hamilton Depression Rating Scale or the Mind Over Mood Anxiety Inventory by Padesky and Greenberger [31,32].

The cognitive behavioral therapist helps to achieve mental and physical improvement mainly by making the patient aware that his or her negative way of thinking about himself or herself, about the disease and about the world leads to the generalization of negative judgments. This, in turn, results in a permanent lowering of a mood that would otherwise lead to a full-blown depression, discouragement, and failure to take care of one’s health. The therapy aims at breaking this sequence and improving the patient’s ability to fight and deal with the disease, while changing the way of thinking about the disease and behaving in response to it. The application of relaxation techniques not only to reduce unpleasant sensations and somatic disorders (muscle pain and tension) but also to reduce emotional tension and anxiety turns out to be effective in cognitive behavioral therapy [32].

### 3.2. Mental Disorders with Somatic Symptoms

Patients presenting mental disorders that have syndromes of somatic problems in their clinical picture are considered a therapeutic group with different expectations. Theoretical assumptions of cognitive behavioral therapy do not fully exhaust the issue of work with this population of patients on somatic symptoms as separate therapeutic techniques, focusing on the diagnosis of the mental illness [14,15].

In the population of people with mental disorders, there is a higher risk of somatic symptoms and falling ill with somatic diseases, which additionally creates in the long run a vicious diagnostic and therapeutic circle and significantly deteriorates the quality of life of the patients. The comorbidity of mental disorders with somatic symptoms complicates the therapeutic process, as the patient is exposed to a higher risk of relapse, higher intensity of manifestations, prolongation of treatment and decrease of therapy effectiveness. Another difficulty is the circumstance in which the patient suffering from, e.g., depressive disorders or anxiety disorders experiences high intensity of physical symptoms (e.g., fatigue, insomnia, appetite disorders, tachycardia or dizziness) and is offered psychotherapy after numerous medical consultations that do not explain the basis of the experienced symptoms [10,22,33]. Therapeutic work will initially focus on accepting the diagnosis of the mental illness. The cognitive behavioral psychotherapist should carefully modify standard therapeutic protocols (activity planning, change of cognitive distortions), adapting them to the individual cases of their patients and the dominant signs that motivate the patient to engage in the therapy [34,35,36,37].

An element of physical sensations, including pain (e.g., so-called masked depressions), which are located in various organs or take the form of suffering, described by the patient as “mental pain”, is often present in the picture of affective disorders. The somatic sensations resulting from these problems are generally blurred and have no precise location, which may give the impression of a comorbid somatic condition, which the patient focuses most of his or her attention on during therapy. Cognitive behavioral therapy is then reduced to a model of working with a depressive patient, the purpose of which is to identify erroneous cognitive patterns and change them in the area of the depressive cognitive triad. The presence of depressive ways of perceiving reality, one’s future and one’s self-image also applies to the image of one’s own illness. During therapy, the patient learns to change his or her way of thinking, also with regard to the somatic symptoms that he or she has so far interpreted in a depressive way (e.g., as a sign of an incurable disease or as nihilistic delusions) [31,38]. Through the use of specific behavioral interventions, the patient can be taught specific skills and behavior patterns that should coexist with adaptation to the disease and its nature, in which somatic and mental manifestations are inseparably intertwined [9,34].

In mental disorders, most often in depression and anxiety disorders, somatic symptoms such as sleep disorders, appetite problems, decreased libido or chronic feeling of fatigue can significantly affect the patient’s functioning. This is reflected in the therapeutic process when working with the patient becomes more difficult, requires more effort or does not bring greater results. Therapeutic techniques (i.e., recording of activity, monitoring of insomnia, daily recording of thoughts) should then be adapted to the patient’s abilities, focusing on an empathic understanding and emotional support so as not to induce feelings of guilt and decline in self-esteem due to reduced abilities that result from the dominant somatic disorders [14,15,31].

### 3.3. Somatic Symptom Disorders

Cognitive behavioral therapy is commonly used in the treatment of most somatization disorders—functional syndromes of organs and systems (e.g., irritable bowel syndrome, tachycardia) and somatic symptom disorders (e.g., hypochondriacal disorders, psychogenic pain)—which is clearly justified by research on its efficacy [39,40]. This group of patients constitutes the population that is most frequently the subject of studies evaluating the effectiveness of applied cognitive behavioral techniques. The reports present exemplary therapeutic techniques, their efficacy and treatment algorithms based on them [7,23]. The key phenomenon in this group of disorders is somatization. It is defined as the process of experiencing mental discomfort in the form of bodily conditions, which the patient interprets as traits of a serious illness. It represents a certain continuum from purely mental phenomena to somatic diseases, in which during the therapeutic process the patient’s automatic thoughts present, i.e., a reflexive look at their illness on the one hand and hypochondriacal delusions about one’s health condition on the other hand [14].

In the case of somatic symptom disorders, patients question or reject medical assurances that there is no basis for the diagnosis. It happens that patients—after undergoing tests, the results of which indicate no diagnosis of a somatic disease—feel short-term reassurance. During the following days, however, doubts about the reliability of the test results and the competence of the doctor grow, and the patient starts looking for another specialist. Patients suffering from somatization disorders have difficulty in accepting the mental background of their illness. In addition, consideration regarding the diagnosis of a mental somatic disease often involves a negative connotation and is mistakenly associated with provoking the disease, which aggravates the patient’s unfavorable mental state [6,14,15,18].

The psychotherapist’s way of communicating with the patient, as early as when making initial contact, is crucial at this point. An important stage of the diagnostic and therapeutic process is a redistribution of the way the patient feels and thinks about his or her disease, the actual risk associated with that and its impact on everyday functioning [2]. Patients often focus on a detailed description of their somatic symptoms and do not allow themselves to think about the possibility of a psychological background of their illness. The patient may use the therapy itself as an attempt to prove its ineffectiveness and thus to deny the psychological background of the disorder and redirect attention to the somatic background only. The described difficulties in the therapeutic relationship are typical for patients who suffer from hypochondriacal disorders. They feel fear of getting sick, giving the impression of being ready for new manifestations of a serious physical illness [41].

The patient with psychogenic somatic disorders experiences genuine suffering, thereby calling him or her a “hypochondriac” arouses aversion to psychotherapeutic care [2]. The *DSM-5* classification [6] takes into account, for the first time, the possibility of a diagnosis of an organic disease coexisting with the patient’s fear for health, in which case the patient’s symptoms are exaggerated. During therapy, the patient with anxiety about his or her health reveals hypochondriacal thoughts, which, according to some authors, have the shape of hyperquantivalent ideas. The patient may accept the lack of grounds for concern, but he or she continues to think about them and follows the same direction [40,42].

In the case of this type of disorder, the patient, during the psychotherapeutic process, reveals a strong desire for the therapist’s support in proving the existence of a somatic condition, while the therapist’s main goal is to help the patient to cope with his or her anxiety. In the conceptualization of health anxiety, particular attention is paid to the secondary gains of the disease, such as being relieved of responsibilities and gaining the privileges of the sick, e.g., care, concern, and attention, all of which are factors that sustain the disease. The aim of the therapy is to break the mechanism of the vicious circle by entering the role of the patient and by paying attention to secondary benefits of the disease from the perspective of the patient and functional disorders, which in this group of patients are comparable to depression and anxiety disorders [43].

An important aspect is to set the goal of the therapy and its duration in an appropriate and realistic way. It is wrong to only question the patient’s belief of having a serious illness. The therapy should aim to try to explain the problem in a more reliable way, which is practically done by undermining beliefs about the disease and building an alternative explanation of the problem. This is done by gathering evidence in favor of an alternative psychological model that will represent a conceptual change from the patient’s disease model. Cognitive behavioral therapy is helpful in making the patient aware of the fact that his or her problem is more related to worrying about the disease—i.e., misinterpretation of the symptoms—and less related to the actual disease. For this purpose, behavioral experiments are used, which focus on showing the effects of selective attention, rumination and examination of the body and not on the realization of the catastrophe. Simple therapeutic techniques for somatoform disorders also include psychoeducation, relaxation, positive suggestion, guided imagery and reattribution training [7,23]. If the patient meets the criteria of mental disorders coexisting with health anxiety, it is important to remember their impact on the therapeutic process and the necessity of their treatment [42] Table 1.

## 4. Conclusions

The increase in the incidence of functional and somatization symptoms, as well as the need for psychotherapeutic care among patients with somatic disorders, suggests that taking the comorbidity of mental and somatic symptoms into account should become a habit in the daily practice of every doctor, psychologist and psychotherapist. The patient not only “feels” somatic disorders, but also “experiences” them. Somatic symptoms that co-occur with mental health problems are primarily disease entities that are not always reflected in disease classifications and clinical subdivisions, and patients experience frustration about ineffective treatment of their complaints, lack of understanding and often even stigmatization.

On the basis of cognitive behavioral psychotherapy, cognitive models, protocols and techniques have been developed that allow the realization of psychotherapeutic effects that are not always applied to the group of patients with somatic symptoms. It should be remembered that before starting therapeutic work, a consideration of the background of the manifestations that the patient reports to the psychotherapist becomes crucial. This dictates case conceptualization and interventions aimed at recognizing and modifying dysfunctional patient beliefs about the disease, as well as the possibilities of its treatment and individual coping. Research on CBT has been widely reported, but it focuses on selected disease entities and does not exhaust all the circumstances of co-occurrence of somatic symptoms and psychological factors.

For all psychosomatic diseases, the ability of an individual to adapt to the disease depends on the nature of cognitive representations developed before the disease. Cognitive representations can be adaptive or maladaptive. The occurrence of the disease as new information juxtaposed with the previously formed image of the world may cause catastrophic thinking and imagining, which intensifies the stress associated with the disease. In the process of cognitive behavioral therapy, the patient, by means of conscious information processing and behavior modification, draws attention to a new interpretation of his or her own situation. Thanks to this, the patient has a chance to achieve an emotional balance and well-being, resulting from the modification of the patient’s views and goals but also through empathic listening to the patient and enabling him or her to express feelings and relieve tension [44,45].

The presented review shows a wide spectrum of diseases in which a relationship between psychological and physical symptoms is observed. It also constitutes an attempt to approach this issue from the perspective of exemplary cognitive behavioral interventions. Adopting such a broad perspective limited the possibility of making a thorough analysis of the types of assessments and therapeutic techniques used and their effectiveness in the discussed disease entities. The authors present this issue to show the magnitude of a problem that is the source of frequent diagnostic dilemmas and difficulties in defining the goals of therapy with patients demonstrating somatic symptoms.

The number of disease entities mentioned suggests that each deserves a separate publication. The development of a future literature review through the use of meta-analysis may yield additional valuable conclusions in evaluating the effectiveness of CBT compared with other therapeutic interventions.

## Figures and Tables

**Table 1:** The relationship between psyche and body in the psychotherapeutic process.

Chronic Somatic Disease	Mental Disorders with Somatic Symptoms	Somatic Symptom Disorders
	**Main Problems**	
psychological reactions caused by physical symptoms and effects of applied treatment frequent examination, pain and hospitalization fear of suffering and death changes in family situation changes in self-image and activity difficulties in accepting a chronic somatic disease	somatic symptoms in the course of psychiatric illness (e.g., insomnia, fatigue, tachycardia) higher risk of somatic symptoms and falling ill with somatic diseases higher risk of relapse somatic symptoms are interpreted in a depressive manner possibility of ignoring the psychological background	experiencing mental discomfort in the form of bodily conditions functional syndromes of organs and systems anxiety about health reveals hypochondriacal thoughts or ideas difficulties in accepting or rejection of psychiatric treatment and psychotherapy making many visits to the primary care physician
**For all patients with somatic symptoms**: Strong focus on physical ailments. Diagnostic dilemma—often impossible to decide which symptoms were the cause and which were the effect. Persistent somatic symptoms worsen the mental state, which in turn leads to the intensification of physical ailments. The possibility of ignoring the participation or background of psychological factors.		
